# Feasibility of Home-Based Spirometry Monitoring as a Decentralized Clinical Trial Component in Patients with Chronic Obstructive Pulmonary Disease and Other Respiratory Diseases

**DOI:** 10.3390/healthcare14182953

**Published:** 2026-09-10

**Authors:** Ye Chan Park, So-Yun Kim, Green Hong, Jang Hee Hong, Dongil Park, Jung Sunwoo

**Affiliations:** 1 Clinical Trials Center, Chungnam National University Hospital, Daejeon 35015, Republic of Korea; dpcks33@naver.com (Y.C.P.); boniii@cnu.ac.kr (J.H.H.); 2 Department of Medical Science, College of Medicine, Chungnam National University, Daejeon 35015, Republic of Korea; 3 Division of Pulmonary, Allergy and Critical Care Medicine, Department of Internal Medicine, Chungnam National School of Medicine, Chungnam National University Hospital, Daejeon 35015, Republic of Korea; soyun3132@cnuh.co.kr (S.-Y.K.); ihongreen@cnuh.co.kr (G.H.); 4 Department of Pharmacology, College of Medicine, Chungnam National University, Daejeon 35015, Republic of Korea

**Keywords:** pulmonary disease, chronic obstructive, spirometry, telemedicine, mobile applications, patient compliance, clinical trial

## Abstract

Introduction: Home-based spirometry has emerged as a potential component of decentralized clinical trials (DCTs) in respiratory medicine, but its real-world feasibility in patients with chronic obstructive pulmonary disease (COPD) and other respiratory diseases remains insufficiently characterized. This study evaluated the feasibility of remote pulmonary function monitoring using a portable spirometer and mobile application in adults with COPD or other respiratory diseases. Methods: This exploratory single-arm study enrolled adults aged 19 years or older with COPD or other respiratory diseases. Participants were asked to perform home-based spirometry at six prespecified time points (Days 15, 29, 43, 57, 71, and 85). Feasibility was assessed using visit-level and participant-level adherence, spirometry quality grades, and patient-reported usability and satisfaction. Participants completing at least 5 of 6 scheduled measurements were classified as the high-adherence group; this cut-off was an operational, study-specific definition. Results: A total of 30 participants were enrolled (19 with chronic obstructive pulmonary disease, 5 with asthma, 2 with asthma–COPD overlap, and 4 with other respiratory conditions), and 23 completed the study. Visit-level completion rates declined over time, from 88.5% on Day 15 to 47.8% on Day 71 and 56.5% on Day 85. Nine participants completed all six measurements, while 13 completed at least five. Portable spirometry quality showed variability across visits, with both high-quality and low-quality measurements observed. Compared with the low-adherence group, the high-adherence group reported significantly higher scores for convenience, communication with the research team, overall satisfaction, willingness to participate in future telemedicine-based trials, device training adequacy, ease of device use, and application usability. Conclusions: Home-based spirometry may be a useful decentralized trial component in patients with COPD and other respiratory diseases; however, the decline in adherence over time and the variability in measurement quality indicate that substantial operational refinement is needed before broader implementation. Because no feasibility threshold was prespecified, no concurrent laboratory spirometry comparison was performed, and the study population was diagnostically heterogeneous, these findings are exploratory and address operational feasibility rather than measurement validity.

## 1. Introduction

Chronic obstructive pulmonary disease (COPD) is a heterogeneous condition characterized by persistent, progressive airflow limitation due to airway and/or alveolar abnormalities, resulting in chronic symptoms such as dyspnea, cough, and sputum production [1]. As of 2019, approximately 400 million people were affected worldwide, and COPD is the leading underlying cause of death among chronic respiratory diseases [2,3].

Spirometry is the objective standard for diagnosing COPD and for assessing the severity of airflow limitation, disease progression, and treatment response [1,4]. Accordingly, international guidelines recommend post-bronchodilator spirometry for accurate diagnosis and emphasize its reproducibility, underscoring the need for reliable and repeated measurements in both clinical and research settings [1,4,5]. How to assess pulmonary function regularly in clinical trials, however, remains an unresolved challenge.

The COVID-19 pandemic exposed the limitations of conventional site-based trials and accelerated interest in decentralized clinical trials [DCTs], which incorporate telehealth visits, electronic consent (eConsent), electronic clinical outcome assessment (eCOA), digital health technologies (DHTs), and home-based monitoring [6,7,8,9,10,11,12]. By reducing travel and visit burden, lowering geographic and socioeconomic barriers, and enabling data collection in real-world settings, DCTs are regarded as a patient-centered model whose role in future trials is expected to grow [8,9,12].

Evidence for remote monitoring in COPD is accumulating. A meta-analysis suggested that telemonitoring may improve emergency department visits, exacerbation-related readmissions and length of stay, mortality, and St. George’s Respiratory Questionnaire scores [13], although a literature review noted that monitoring duration, technologies, and feedback methods vary across studies, and that higher data-transmission frequency was associated with reduced adherence [14]. Recent studies of home spirometry in interstitial lung disease (ILD) and other chronic respiratory diseases have reported high adherence and acceptability—for example, 77% of ILD patients met spirometry adherence on ≥70% of study days—and confirmed home spirometry as an accurate, feasible method [15,16]. Nonetheless, challenges such as measurement frequency, repeated-measurement methodology, patient education, device and connectivity issues, and greater data variability than hospital-based spirometry remain, leading some to argue that further validation is needed before home spirometry can serve as a primary trial endpoint [17].

Against this background, this study aimed to evaluate the feasibility of remote pulmonary function monitoring using a portable spirometer and a mobile application within a decentralized clinical trial in adults with COPD or other respiratory diseases, and to identify operational issues arising during real-world implementation.

## 2. Materials and Methods

### 2.1. Study Design

The present study was an exploratory, single-arm study conducted to evaluate the feasibility of remote monitoring incorporating DCT components in patients with COPD and other respiratory diseases. No comparator arm was established, and randomization was not performed. Screening was performed within the hospital, after which remote monitoring was conducted in out-of-hospital settings using a portable spirometer, with no restrictions placed on the out-of-hospital environment. Study participants were provided with a portable spirometer and were instructed to use the device at prespecified time points defined in the protocol (Days 15, 29, 43, 57, 71, and 85 following study enrollment, with a permissible window of ±7 days). In addition, a survey on study participation experience was administered at the end of the study. The measurement time points were set at approximately 2-week intervals over a 12-week period. This schedule was designed to allow measurements to be performed within the average interval between outpatient visits while minimizing participant burden.

A portable, Bluetooth-connected spirometer (Spirokit, TR Co., Ltd., Daejeon, Republic of Korea) was used for pulmonary function measurements [18].

### 2.2. Study Objectives

The primary objective of the present study was to assess the feasibility of remote monitoring incorporating decentralized clinical trial components. In addition, the study aimed to identify issues that may arise during the remote monitoring process and to evaluate participants’ experiences with, and satisfaction regarding, study participation. Because the study was exploratory in nature, no formal feasibility threshold—such as a minimum per-visit completion rate, a minimum number of measurements per participant, or a target proportion of acceptable-quality measurements—was prespecified in the protocol, and no formal success criterion was defined. Feasibility outcomes were therefore summarized descriptively, and all conclusions regarding feasibility are to be regarded as exploratory and hypothesis-generating. Spirometry was selected as the monitoring modality because it is the objective standard for assessing airflow limitation and is the endpoint most frequently required in respiratory clinical trials. The intention was to determine whether this trial-relevant measurement can be obtained remotely within a decentralized design.

### 2.3. Participants

Eligible participants were adults aged 19 years or older who, at the time of screening, had a diagnosis of COPD or had been receiving care at Chungnam National University Hospital for a respiratory disease for at least six months. Key inclusion criteria comprised the ability to provide written informed consent, the capacity for self-care and independent ambulation, and an Eastern Cooperative Oncology Group Performance Status of 2 or lower. Conversely, individuals were excluded if they were unable to operate a portable device, had a scheduled procedure that might influence pulmonary function test results, or had been diagnosed with an acute exacerbation of COPD or shown evidence of worsening of an underlying respiratory disease within the preceding month. The specific underlying respiratory diagnosis of each participant was recorded at screening, and each participant was assigned to a single primary diagnostic category; the diagnostic distribution of the enrolled population is presented in Table 1.

### 2.4. Adherence and Feasibility Outcomes

The adherence outcomes comprised the per-visit completion rate of portable spirometry and the total number of measurements performed per participant. In addition, adherence was compared at the participant level. No absolute threshold has been established for the completion rate of portable spirometry. The present study therefore adopted a cut-off of 80%, which has been used in other studies either as a criterion for exclusion from analysis because of insufficient protocol adherence or as a threshold for evaluating compliance [19,20]. Accordingly, participants who completed at least 5 of the 6 prespecified measurement time points (83.3%) were categorized as the high-adherence group. This cut-off constitutes an operational, study-specific definition adopted to permit descriptive comparison between participants. No validated threshold for defining adequate adherence to home-based spirometry has been established, and the cut-off applied here should therefore not be interpreted as a clinically validated standard.

### 2.5. Spirometry Quality and Outcomes

The quality of the portable spirometry results was graded as A, B, C, D, or F, with progression from A toward F indicating decreasing suitability for the interpretation of pulmonary function [Supplementary Table S1]. During the study, quality grades were assigned automatically by the algorithm embedded in the device and its paired mobile application, according to the criteria summarized in Supplementary Table S1. Individual flow-volume curves were not re-reviewed or re-graded by an investigator.

### 2.6. Patient-Reported Usability and Satisfaction Outcomes

Patient-reported usability and satisfaction were evaluated using five items related to the DCT, four items related to the portable spirometer, and the aggregate total score across all survey items [Supplementary Table S2]. The questionnaire was administered on a single occasion, at the end-of-study visit, after the entire follow-up period had been completed; no interim administration was performed. Satisfaction was therefore measured after adherence behavior had already occurred.

### 2.7. Data Collection and Data Management

Pulmonary function measurement data obtained with the portable spirometer were collected as records automatically registered through the linkage between the portable spirometer and a smartphone application. At the time of screening, demographic information of the participants was collected through in-person visits, and at the end-of-study time point, survey data were collected likewise through in-person visits. With respect to pulmonary function measurement data acquired via the portable spirometer, missing data attributable to non-performance and those attributable to study withdrawal were not treated as equivalent in data interpretation; data missing due to withdrawal were interpreted as reflecting the loss of any further opportunity to perform pulmonary function testing, and accordingly, time points following the withdrawal were not incorporated into the adherence assessment. When a scheduled measurement was not performed, the research team contacted the participant and recorded the reason for non-performance. Reasons were captured only as the broad category of participant personal circumstances and were not collected in a structured or itemized format. Clinical events occurring during the study period—including acute exacerbations, hospitalizations, and changes in respiratory maintenance treatment—were collected as adverse events.

### 2.8. Statistical Analysis

All statistical analyses were performed using R statistical software (R Foundation for Statistical Computing, Vienna, Austria; version 4.5.3). Categorical variables were presented as frequencies and percentages, whereas continuous variables were summarized as mean ± standard deviation or median with interquartile range, depending on whether the assumption of normality was met.

Adherence to portable spirometry was evaluated based on the number of completed measurements out of the six prespecified visits, and participants who completed five or more measurements were classified into the high-adherence group, while those who completed fewer than five were classified into the low-adherence group. Per-visit completion rates were calculated using a dynamic denominator: at each scheduled visit, the denominator comprised only those participants who remained in the study at that visit and were therefore still eligible to perform the measurement. A consequence of this convention is that the denominator shrinks over time and that completion rates at later visits are conditional on continued study participation; they are therefore difficult to compare directly with the rates observed at earlier visits.

For the comparison of survey responses between the two groups, the Wilcoxon rank-sum test was applied. Effect sizes were calculated using Cliff’s delta, and adjustment for multiple comparisons was performed using the Benjamini–Hochberg false discovery rate (FDR) procedure; adjusted *p*-values are reported as pFDR. The magnitude of Cliff’s δ was interpreted using the following thresholds: |δ| < 0.15 as negligible, 0.15 ≤ |δ| < 0.33 as small, 0.33 ≤ |δ| < 0.47 as medium, and |δ| ≥ 0.47 as large [21]. All tests were two-sided, and statistical significance was defined as *p* < 0.05.

## 3. Results

### 3.1. Study Population and Demographics

**Figure 1:**
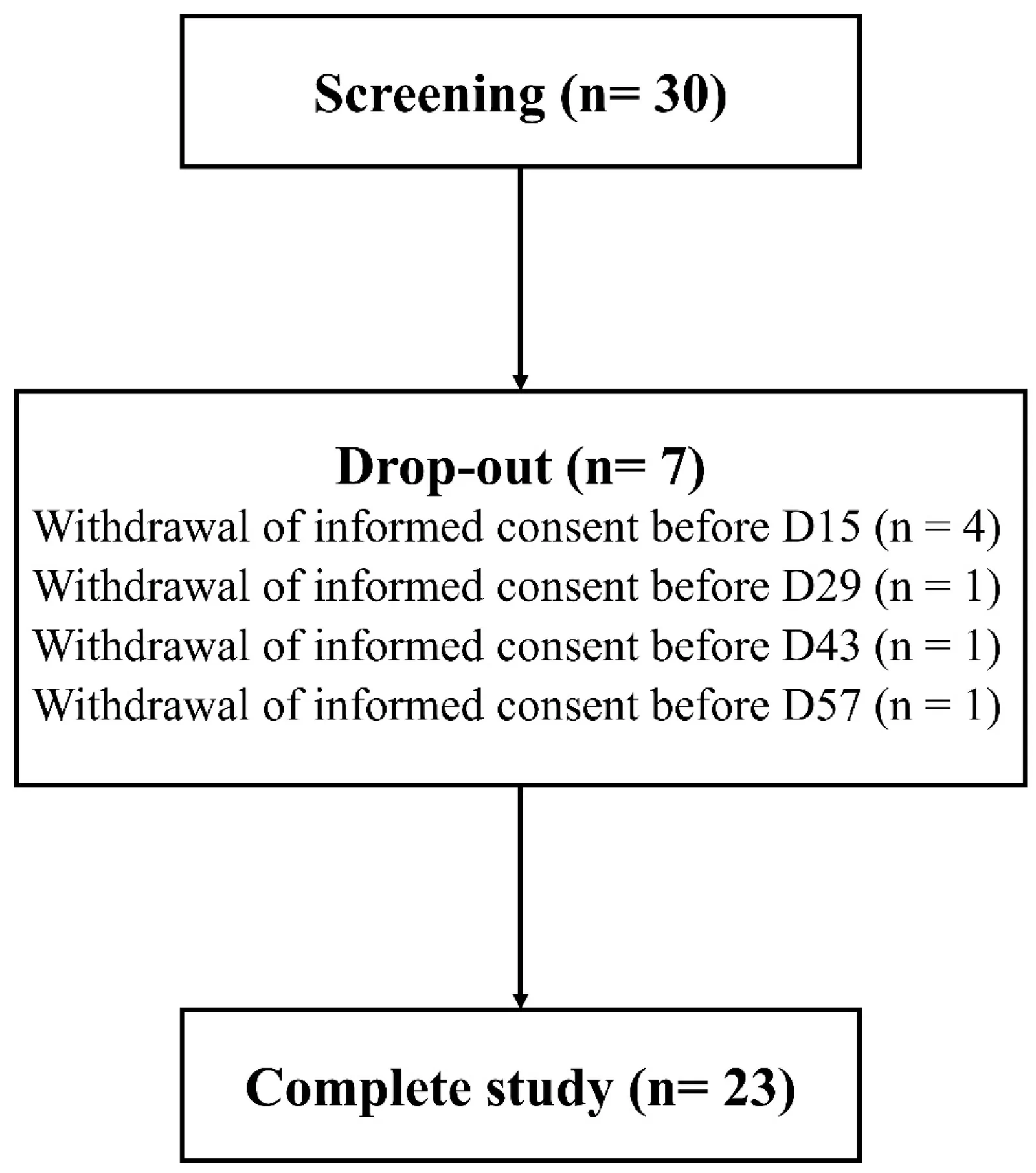
Flow diagram of study population.

The study was conducted at Chungnam National University Hospital from 26 August 2024 to 17 March 2026. A total of 30 participants were enrolled in the study. Four participants withdrew consent before the first scheduled home spirometry assessment (D15), leaving 26 participants eligible for the first remote measurement. An additional 3 participants subsequently withdrew consent during the follow-up period; thus, 23 participants (76.7%) completed the study, and 7 (23.3%) discontinued in total [Figure 1]. With respect to the underlying respiratory diagnosis, 19 participants had COPD, 5 had asthma, and 2 had asthma–COPD overlap, while the remaining 4 participants had other respiratory conditions (atelectasis, lung ground-glass opacity, obstructive sleep apnea, and pulmonary tuberculosis; 1 participant each). The mean age (±SD) of the participants was 63.5 (±9.6) years, with 24 men (80%) and 6 women (20%), and the mean body mass index (±SD) was 24.7 (±4.6) kg/m^2^. With respect to smoking status, 7 participants (23%) were non-smokers, 13 (43%) were current smokers, and 10 (33%) were former smokers, while regarding alcohol consumption, 23 (77%) were non-drinkers and 7 (23%) were current drinkers. Other baseline characteristics are summarized in Table 1.

A total of 30 participants were screened, of whom 7 discontinued during the study. The reason for discontinuation in all cases was voluntary withdrawal of informed consent, with the time points of discontinuation as follows: 4 participants prior to Day 15 (D15), 1 prior to Day 29 (D29), 1 prior to Day 43 (D43), and 1 prior to Day 57 (D57). Ultimately, 23 participants completed the study.

### 3.2. Adherence and Feasibility Outcomes

**Figure 2:**
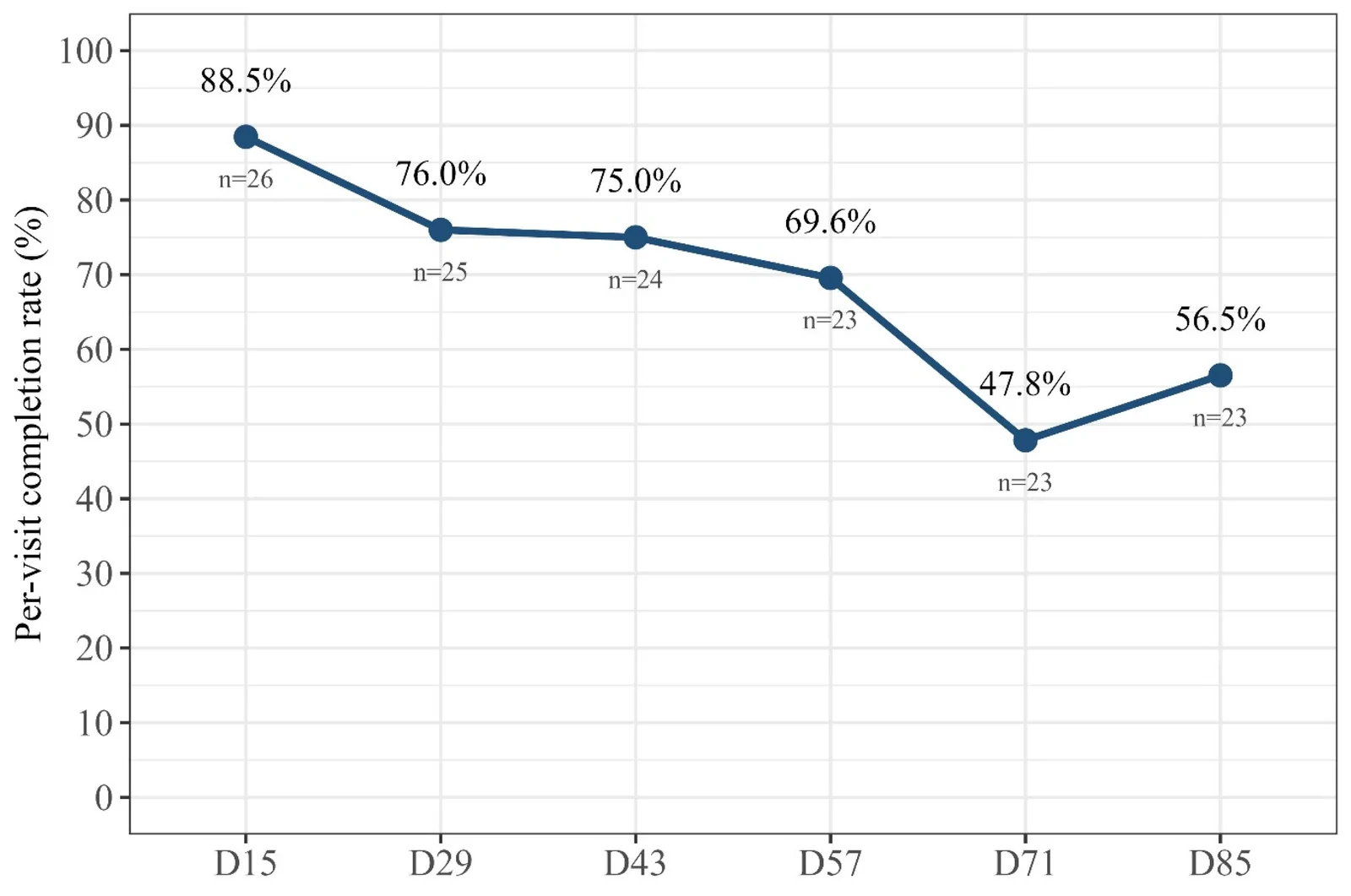
Follow-up spirometry completion over time.

The per-visit completion rates were as follows: at D15, 23 of 26 scheduled participants performed measurements (88.5%); at D29, 19 of 25 (76.0%); at D43, 18 of 24 (75.0%); at D57, 16 of 23 (69.6%); at D71, 11 of 23 (47.8%); and at D85, 13 of 23 (56.5%). Regarding the total number of measurements performed per participant (out of a maximum of six), 9 of the 26 participants (34.6%) completed all six prespecified measurements. Five measurements were performed by 4 participants (15.4%), four by 2 (7.7%), three by 2 (7.7%), two by 4 (15.4%), and one by 4 (15.4%), while 1 participant (3.8%) did not perform any measurements [Table 2, Figure 2]. Because per-visit completion rates were calculated using as the denominator only those participants who remained in the study at each visit, the completion rates at later visits are conditional on continued study participation and are therefore difficult to compare directly with those at earlier visits. For measurements that were not performed, the reason recorded by the research team was, in every instance in which a reason could be obtained, the participant’s personal circumstances; more specific reasons were not systematically documented.

This figure presents the per-visit completion rate (%) of follow-up spirometry performed at each scheduled visit (D15, D29, D43, D57, D71, and D85). Above each data point, the completion rate (%) for the corresponding visit is shown, while the number of participants scheduled to undergo spirometry at that visit (*n*) is indicated below. The completion rate (%) was calculated as the number of participants who completed the measurement at each scheduled visit divided by the number of participants scheduled to undergo spirometry at that visit, multiplied by 100.

### 3.3. Spirometry Quality Outcomes

Across all time points, Grade A measurements ranged from 15.8% to 43.5%, Grade B stayed at or below 9.1%, and lower-quality grades (C, D and F) were consistently present, without a clear trend over time. The full grade distribution at each visit is shown in Supplementary Table S3.

Participants were classified into the A/B group (*n* = 10) or the C/D/F group (*n* = 11) based on the grade of their first performed measurement. Both groups completed all measurements at D15 (100%). Thereafter, completion declined more markedly in the A/B group, reaching 20% (2/10) at D71, whereas the C/D/F group maintained rates of 81.8–90.9%. The between-group difference was statistically significant at D71 (20.0% vs. 81.8%, *p* = 0.01) [Table 3].

### 3.4. Patient-Reported Usability and Satisfaction Outcomes

Across all items, responses in the high-adherence group were statistically significantly higher than those in the low-adherence group. For Q1, which addressed the convenience of out-of-hospital spirometry, the median in the high-adherence group was 4.00 (IQR 0), significantly higher than the 2.00 (1.00) observed in the low-adherence group (*p* < 0.001, pFDR = 0.001, Cliff’s δ = 0.846), with the effect size classified as large. A significant difference was likewise observed for Q2, which addressed the ease of communication with the research team, with medians of 4.00 (0) in the high-adherence group and 2.50 (2.00) in the low-adherence group (*p* = 0.007, pFDR = 0.009, δ = 0.631). Significant between-group differences were also observed for overall satisfaction with remote monitoring (Q3), willingness to participate in future telemedicine-based clinical trials (Q4), and willingness to recommend such trials to others (Q5), with medians of 4.00 in the high-adherence group and 2.00 in the low-adherence group across all three items (*p* = 0.001, pFDR = 0.001, 0.002, and 0.002; δ = 0.815, 0.8, and 0.762, respectively). Notably, in the low-adherence group, 70.0% (7/10) of participants responded to each of Q3, Q4, and Q5, whereas in the high-adherence group, no “dissatisfied” responses were recorded for these items.

With regard to items related to the use of the portable spirometer, a significant difference was observed for the adequacy of device-use training (Q6), with medians of 4.00 (0) in the high-adherence group versus 2.50 (2.00) in the low-adherence group (*p* = 0.008, pFDR = 0.009, δ = 0.608), and significant between-group differences were also noted for the ease of device use (Q7) and the ease of mobile application use (Q8) (*p* = 0.001, pFDR = 0.001, δ = 0.815). For Q9, which addressed the appropriateness of a biweekly measurement schedule, the median was 4.00 in the high-adherence group and 2.00 in the low-adherence group, also indicating a significant difference (*p* = 0.012, pFDR = 0.012, δ = 0.600).

**Figure 3:**
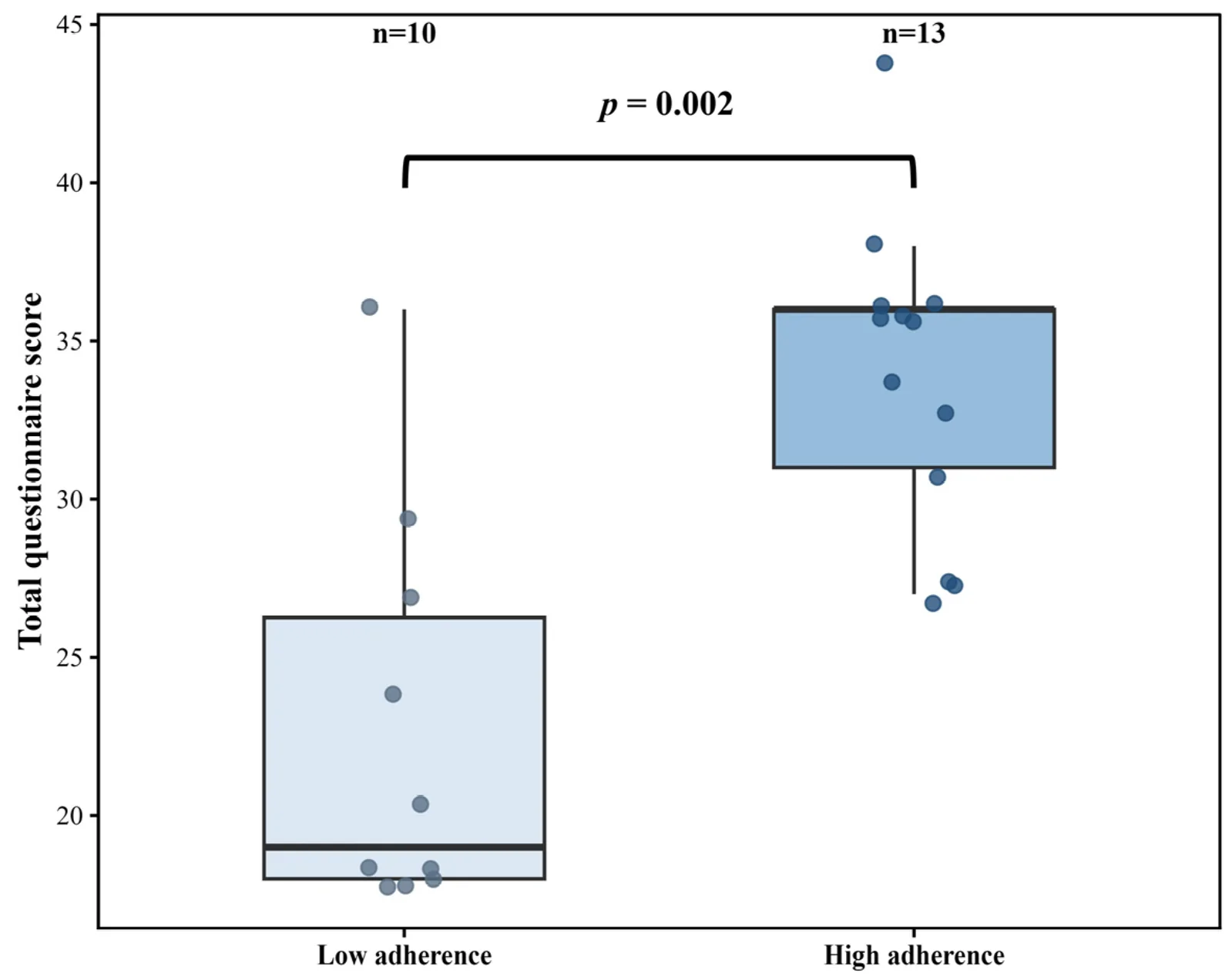
Comparison of total questionnaire scores according to adherence group. Light blue indicates the low adherence group and dark blue indicates the high adherence group.

The total score, obtained by summing the nine items, was 36.00 (5.00) points in the high-adherence group and 19.00 (9.00) points in the low-adherence group, with a significant difference between groups (*p* = 0.001, pFDR = 0.002, δ = 0.800) and a large effect size [Table 4, Figure 3].

Box plot comparing the total questionnaire score between the low-adherence (*n* = 10) and high-adherence (*n* = 13) groups. The box represents the interquartile range (IQR), and the bold horizontal line within the box indicates the median. The whiskers extending above and below the box correspond to the minimum and maximum values within 1.5 × IQR, and the individual dots represent the observed values for each participant. Between-group comparison was performed using the Wilcoxon rank-sum test, and the reported *p*-value (pFDR) was adjusted for multiple comparisons using the Benjamini–Hochberg procedure to control the false discovery rate.

Abbreviations: IQR, interquartile range; App, mobile application; pFDR, *p*-value adjusted using the Benjamini–Hochberg false discovery rate procedure.

In a supplementary analysis restricted to participants with an evaluable first-measurement quality grade, end-of-study questionnaire scores did not differ significantly between the two groups. The mean total score was 28.0 ± 8.0 in the A/B group (*n* = 10) and 31.9 ± 6.7 in the C/D/F group (*n* = 11) (*p* = 0.24) [Supplementary Table S4].

## 4. Discussion

The completion rate of home-based spirometry declined from 88.5% on Day 15 to 47.8% on Day 71, indicating that the principal challenge lies not in providing a device but in sustaining long-term adherence. Although digital monitoring showed high initial acceptability, attrition increased over time as repeated self-measurements were required, for reasons that could not be determined in the present study. Because the reasons for missed measurements were recorded only as unspecified personal circumstances and were not collected in a structured manner, the specific drivers of attrition could not be identified; participant fatigue, measurement burden, technical inconvenience, and interference with daily life are plausible contributors, but they remain hypotheses rather than observed reasons. A similar decline has been reported for weekly home spirometry in idiopathic pulmonary fibrosis (IPF) [22]. No acute exacerbations, hospitalizations, or adjustments to respiratory treatment occurred during the follow-up period.

The high-adherence group also reported significantly higher overall satisfaction than the low-adherence group, suggesting that success depends less on clinical characteristics or medical necessity than on the overall user experience—perceived convenience, ease of using the application, communication with the research team, and acceptability of the measurement schedule. This is consistent with prior work: in IPF and ILD, home spirometry was generally viewed as convenient because it reduced hospital visits, enabled more frequent assessment, and helped patients understand and feel in control of their condition [23,24]. Conversely, mean adherence to a twice-weekly protocol was only 53% among ILD patients in rural areas, showing that measurement frequency itself influences adherence [25]. Together, these findings suggest that the adoption of digital tools may depend not only on the technology itself but also on usability, participant understanding, support systems, and feedback structures.

This association must nevertheless be interpreted with caution. The satisfaction questionnaire was administered only once, at the end of follow-up, and therefore after adherence behavior had already occurred. The relationship between satisfaction and adherence is consequently cross-sectional, and its direction cannot be determined from these data: a favorable user experience may have promoted adherence, participants who adhered may have appraised the experience more favorably in retrospect, or both processes may have operated together. Prospective or repeated measurement of usability and satisfaction during follow-up would be required to establish temporal ordering.

Measurement quality in the present study was more variable than in previous reports of home spirometry. Grade A measurements were obtained at some time points, but quality grades were distributed unevenly and lower-quality measurements occurred. By contrast, a clinical service–based ILD study reported that 85.7% of home spirometry attempts over three months met ERS/ATS acceptability criteria, suggesting that adequate quality is achievable at home when appropriate support is in place [15]. The variability we observed therefore appears to reflect operational limitations—insufficiently refined user training and the lack of a repeated-measurement design, real-time quality feedback, and acceptable quality thresholds.

The manner in which quality was determined also differs between studies and warrants consideration: in the present study grades were assigned automatically by the device algorithm without investigator adjudication, whereas the comparator study applied ERS/ATS acceptability criteria within a supervised clinical service. Differences in how acceptability was ascertained may therefore contribute to the apparent difference in data quality. An unexpected pattern was also observed. Participants whose first measurement was graded A or B subsequently discontinued measurement more rapidly than those whose first measurement was graded C, D, or F, and the difference was significant at D71 (20.0% vs. 81.8%, *p* = 0.01). This suggests that participants who obtained an acceptable result at the outset may have perceived little additional benefit in repeating a test that had already succeeded and may therefore have been more susceptible to protocol fatigue, whereas participants with initially poor grades may have continued in an attempt to obtain a satisfactory measurement. However, given the small number of participants in this study, confirmation in a larger cohort is needed.

These observations point to specific operational remedies. The mobile application could provide immediate feedback after each maneuver and, when repeated Grade D or F measurements are detected, prompt the participant to repeat the test, display brief corrective instructions, or trigger contact from the research team. Embedding such real-time quality control, together with periodic re-training, is likely to be more effective than initial training alone in maintaining measurement quality over time.

Patients with COPD often face barriers to conventional in-person trials owing to symptom exacerbations, limited mobility, advanced age, and the burden of repeated visits. Home-based spirometry can ease this burden while enabling repeated physiological data collection in real-world settings. Comparable studies report that home spirometry reduces travel burden, allows more frequent tracking of lung function, and may support earlier recognition of disease changes and better self-management [15,23,24], and a recent DCT review notes its potential to reduce geographic barriers and improve the inclusiveness and efficiency of research participation [26]. Our findings suggest that portable spirometry may have a role in supporting more patient-centered and decentralized approaches in respiratory trials, although further validation in larger and more rigorously designed studies is needed.

This study has several limitations. As an exploratory single-arm study without randomization or a control group, it could not assess the efficacy or superiority of home-based monitoring. The small sample size limits generalizability. Most importantly, home-based measurements were not compared with concurrent hospital-based laboratory spirometry, and no agreement or validation analysis was undertaken. The present study therefore evaluates the operational feasibility of home-based spirometry—whether participants can and will perform the test remotely and whether the associated workflow is implementable within a decentralized trial—rather than the measurement validity or accuracy of the values obtained. In addition, the study population was diagnostically heterogeneous. Disease-specific differences in symptom burden, exacerbation frequency, familiarity with spirometry, and expected within-patient variability may affect both adherence and test performance.

A further limitation is that no feasibility threshold was prespecified, so the interpretation of the observed completion rates and quality distribution is necessarily descriptive. Finally, home-environment factors such as device unfamiliarity, connectivity errors, and transmission difficulties may have affected data quality independently of clinical status. Because detailed clinical characterization was not obtained in this study, adherence to the use of the portable spirometer according to the clinical characteristics of the recruited patient group could not be determined, and overall treatment adherence was not assessed. In addition, comorbid conditions such as depression and cognitive impairment were not recorded. Some participants nevertheless had uncontrolled hypertension and an elevated heart rate, raising the possibility that treatment adherence was low in part of the sample. Because enrollment extended from August to March, seasonal factors such as influenza and winter exacerbations may also have affected measurement performance. Although no technical problems with the device were reported, unrecorded and unreported participant factors—such as impaired vision or discomfort too mild to prompt a hospital visit—may still have affected adherence. The reasons for discontinuation were recorded only as voluntary withdrawal of consent, and no comparison of baseline characteristics between participants who withdrew and those who completed the study was performed. In addition, the reasons for voluntary withdrawal of consent were not collected, and it is possible that participants who discontinued had more severe disease at baseline. It should also be noted that the day-to-day variability of spirometric parameters is low in COPD, in contrast to asthma, which may have limited the perceived value of repeated self-measurement and thereby reduced motivation. Finally, in the absence of a comparator arm or of concurrent hospital-based spirometry, it is not possible to determine whether the observed decline in adherence reflects the device and the monitoring procedure themselves, a study effect, or external factors. These findings may also vary depending on the study setting.

All 30 participants were enrolled at a single tertiary center, and patients who consented to a technology-based trial at a university hospital are likely to have been more familiar with, and more receptive to, digital devices than the wider population of patients with respiratory disease. In a larger, multicenter population that includes older individuals, those with lower digital literacy, and those with limited access to smartphones or to stable connectivity, both adherence and measurement quality may be lower than observed here. Future studies with larger, controlled designs, more complete clinical characterization, and refined quality-management protocols are warranted to confirm these observations.

## 5. Conclusions

The present study sought to examine whether home-based pulmonary function monitoring using a portable spirometer and a mobile application could be feasibly implemented as a component of a decentralized clinical trial in patients with COPD and other respiratory diseases. Home spirometry showed relatively high completion rates during the early phase of the study, and favorable satisfaction and usability were observed among some participants, suggesting potential usefulness as a remote data-collection approach. Nevertheless, adherence tended to decline over the course of follow-up and variability in measurement quality was observed, indicating that ensuring long-term operational stability and data reliability remains a key challenge. Several refinements therefore appear necessary before home-based monitoring can serve as a core assessment tool in clinical trials. These include optimization of the measurement frequency, reinforcement of user training, and improvement of technical support and feedback systems. Validation against hospital-based standard spirometry is also required. Taken together, home spirometry may offer advantages as a decentralized data-collection approach in respiratory clinical trials. Its broader use, however, will require improvements in participant support, training, measurement protocols, and quality-control procedures. These conclusions should be regarded as exploratory. No feasibility threshold was prespecified, no comparison with concurrent hospital-based laboratory spirometry was undertaken, and the study population was diagnostically heterogeneous. The present findings therefore speak to the operational feasibility of home-based spirometry as a decentralized trial component, and do not establish the measurement validity of home-derived pulmonary function values; formal validation against standard laboratory spirometry remains a necessary next step.

## Figures and Tables

**Table 1:** Study participant demographics.

Variable	Total (*n* = 30)	Low Adherence Group (*n* = 10)	High Adherence Group (*n* = 13)
Age (years)	63.5 ± 9.6 (39.0–78.0)	67.4 ± 7.2 (55.0–78.0)	60.2 ± 9.0 (45.0–74.0)
Sex			
Male	24 (80%)	8 (80%)	10 (77%)
Female	6 (20%)	2 (20%)	3 (23%)
Height (cm)	166.3 ± 7.5 (149.1–184.0)	165.6 ± 7.4 (154.0–176.0)	166.2 ± 6.9 (149.1–173.0)
Weight (kg)	68.8 ± 17.1 (54.0–133.0)	62.5 ± 6.7 (54.4–74.5)	69.2 ± 15.1 (54.0–110.0)
Body mass index (kg/m^2^)	24.7 ± 4.6 (18.4–39.2)	22.8 ± 2.6 (19.0–28.5)	25.1 ± 4.8 (18.4–36.7)
Smoking status			
No (Non-smoker)	7 (23%)	2 (20%)	3 (23%)
Yes (Current smoker)	13 (43%)	4 (40%)	6 (46%)
Former smoker	10 (33%)	4 (40%)	4 (31%)
Alcohol consumption			
No (Non-drinker)	23 (77%)	7 (70%)	11 (85%)
Yes (Current drinker)	7 (23%)	3 (30%)	2 (15%)
Disease (*n*)			
COPD	19	8	7
Asthma	5	1	3
Asthma–COPD overlap	2	1	1
Atelectasis	1	0	0
Lung ground-glass opacity	1	0	1
Obstructive sleep apnea	1	0	0
Pulmonary tuberculosis	1	0	1
Systolic blood pressure (mmHg)	131.6 ± 12.9 (98.0–154.0)	130.3 ± 11.6 (110.0–144.0)	132.9 ± 11.7 (112.0–153.0)
Diastolic blood pressure (mmHg)	78.4 ± 11.1 (58.0–106.0)	75.1 ± 9.4 (58.0–92.0)	79.3 ± 11.1 (62.0–101.0)
Pulse rate (beats/min)	79.6 ± 11.4 (56.0–110.0)	72.8 ± 8.8 (56.0–84.0)	81.6 ± 10.2 (65.0–97.0)
Respiratory rate (breaths/min)	17.2 ± 2.1 (14.0–24.0)	16.6 ± 1.3 (14.0–19.0)	16.9 ± 2.1 (15.0–23.0)
Body temperature (°C)	36.7 ± 0.1 (36.5–36.9)	36.7 ± 0.1 (36.5–36.8)	36.7 ± 0.1 (36.5–36.8)

Note: Data are presented as mean ± standard deviation (range) for continuous variables and as *n* (%) for categorical variables. Percentages may not sum to 100% due to rounding. Disease categories reflect the underlying respiratory diagnosis recorded at screening; each participant was assigned to a single primary diagnostic category.

**Table 2:** Per-visit performance status and distribution of the total number of measurements per participant.

	Expected *n*	Performed *n* (%)
Per-visit performance		
D15	26	23 (88.5%)
D29	25	19 (76.0%)
D43	24	18 (75.0%)
D57	23	16 (69.6%)
D71	23	11 (47.8%)
D85	23	13 (56.5%)
Total number of measurements performed per participant (range: 0–6, total *n* = 26)		*n* (%)
0 times		1 (3.8%)
1 time		4 (15.4%)
2 times		4 (15.4%)
3 times		2 (7.7%)
4 times		2 (7.7%)
5 times		4 (15.4%)
All performed		9 (34.6%)

Note: Expected *n* indicates the number of participants who were scheduled and eligible for the portable spirometry assessment at each visit; participants who had discontinued or were lost to follow-up prior to the scheduled visit were excluded from the denominator. Performed *n* (%) represents the number and proportion of participants who completed the assessment among those expected. The lower panel summarizes the cumulative number of the portable spirometry measurements completed per participant across all six scheduled visits (D15–D85), based on the full analysis set (*n* = 26). Percentages may not sum to 100% due to rounding.

**Table 3:** Comparison of measurement completion rates over time between first-measurement quality grade groups.

Visit Period	A/B Group	C/D/F Group	*p* Value
D15	10/10 (100%)	11/11 (100%)	-
D29	7/10 (70%)	9/11 (81.8%)	0.64
D43	7/10 (70%)	10/11 (90.9%)	0.31
D57	6/10 (60%)	10/11 (90.9%)	0.15
D71	2/10 (20%)	9/11 (81.8%)	0.01
D85	4/10 (40%)	9/11 (81.8%)	0.08

Note: Participants were classified into the A/B group (*n* = 10) or the C/D/F group (*n* = 11) based on the quality grade of their first performed measurement. Data are presented as the number of participants who completed measurement/total number in each group (%). *p* values were calculated using Fisher’s exact test comparing the two groups at each visit; the comparison at D15 was not performed because both groups achieved 100% completion. Of the 23 participants who performed home spirometry at Day 15, 21 had both an evaluable first-measurement quality grade and available questionnaire data and were therefore included in the supplementary comparison according to first-measurement quality grade.

**Table 4:** Comparison of questionnaire responses between high and low adherence groups.

Questions	Group	*n*	Score Distribution, *n* (%)					Median (IQR)	*p*	pFDR	δ
			1	2	3	4	5				
Q1. Was the out-of-hospital spirometry testing convenient?	High	13	0	0 (0.0)	3 (23.1)	8 (61.5)	2 (15.4)	4.00 (0)	<0.001	0.001	0.846
	Low	10	0	7 (70.0)	2 (20.0)	1 (10.0)	0 (0.0)	2.00 (1.00)			
Q2. Was it easy to communicate with the research team during the study period?	High	13	0	0 (0.0)	3 (23.1)	8 (61.5)	2 (15.4)	4.00 (0)	0.007	0.009	0.631
	Low	10	0	5 (50.0)	2 (20.0)	3 (30.0)	0 (0.0)	2.50 (2.00)			
Q3. Overall, were you satisfied with the remote monitoring via phone calls and the mobile application (App)?	High	13	0	0 (0.0)	4 (30.8)	8 (61.5)	1 (7.7)	4.00 (1.00)	0.001	0.001	0.815
	Low	10	0	7 (70.0)	2 (20.0)	1 (10.0)	0 (0.0)	2.00 (1.00)			
Q4. Are you willing to participate in future clinical trials that include telemedicine components?	High	13	0	0 (0.0)	5 (38.5)	6 (46.2)	2 (15.4)	4.00 (1.00)	0.001	0.002	0.8
	Low	10	0	7 (70.0)	2 (20.0)	1 (10.0)	0 (0.0)	2.00 (1.00)			
Q5. Would you be willing to recommend clinical trials with telemedicine components to others?	High	13	0	0 (0.0)	6 (46.2)	7 (53.8)	0 (0.0)	4.00 (1.00)	0.001	0.002	0.762
	Low	10	0	7 (70.0)	2 (20.0)	1 (10.0)	0 (0.0)	2.00 (1.00)			
Q6. Was the training provided for the portable spirometer adequate?	High	13	0	0 (0.0)	3 (23.1)	9 (69.2)	1 (7.7)	4.00 (0)	0.008	0.009	0.608
	Low	10	0	5 (50.0)	2 (20.0)	3 (30.0)	0 (0.0)	2.50 (2.00)			
Q7. Was the portable spirometer easy to use?	High	13	0	0 (0.0)	4 (30.8)	8 (61.5)	1 (7.7)	4.00 (1.00)	0.001	0.001	0.815
	Low	10	0	7 (70.0)	2 (20.0)	1 (10.0)	0 (0.0)	2.00 (1.00)			
Q8. Was the mobile application (App) easy to use?	High	13	0	0 (0.0)	4 (30.8)	8 (61.5)	1 (7.7)	4.00 (1.00)	0.001	0.001	0.815
	Low	10	0	7 (70.0)	2 (20.0)	1 (10.0)	0 (0.0)	2.00 (1.00)			
Q9. Was the biweekly spirometry testing frequency using the portable device appropriate?	High	13	0	1 (7.7)	4 (30.8)	7 (53.8)	1 (7.7)	4.00 (1.00)	0.012	0.012	0.6
	Low	10	0	6 (60.0)	2 (20.0)	2 (20.0)	0 (0.0)	2.00 (1.00)			
Total score	High	13	36.00 (5.00)				0.001		0.002		0.8
	Low	10	19.00 (9.00)								

Note: Data are presented as *n* (%) for each response score and as median (interquartile range). Each item was rated on a five-point scale (1 = very dissatisfied, 2 = dissatisfied, 3 = neutral, 4 = satisfied, 5 = very satisfied), with higher scores indicating more favorable responses. The high-adherence group was defined as participants who performed home-based spirometry on five or more occasions during the study period, whereas the low-adherence group was defined as those who performed it on fewer than five occasions. Between-group comparisons were conducted using the Wilcoxon rank-sum test. pFDR denotes the *p*-value adjusted for multiple comparisons using the Benjamini–Hochberg procedure. δ refers to the effect size, calculated as Cliff’s delta (δ). The total score represents the sum of the nine items and is presented as median (interquartile range).

## Data Availability

The data presented in this study are available on request from the corresponding author. The data are not publicly available due to privacy restrictions, as they contain information that could compromise the privacy of the study participants.

## Supplementary Materials

The following supporting information can be downloaded at: https://www.mdpi.com/article/10.3390/healthcare14182953/s1, Supplementary Table S1: Pulmonary function test grades and definitions; Supplementary Table S2: Patient Satisfaction Questionnaire; Supplementary Table S3: Portable Spirometry Quality; Supplementary Table S4: Questionnaire scores according to the quality grade of the first performed home spirometry measurement.

## Clinical Trial Registration

This trial was registered at the Clinical Research Information Service (CRIS; KCT0011124).
