# Supplementary material for: Feasibility of Home-Based Spirometry Monitoring as a Decentralized Clinical Trial Component in Patients with Chronic Obstructive Pulmonary Disease and Other Respiratory Diseases
Source: Healthcare (Basel). 2026 Sep 10;14(18):2953. doi: 10.3390/healthcare14182953 (PMC13606866; doi:10.3390/healthcare14182953)
Supplement: Supplementary file 1 [file healthcare-14-02953-s001.zip › healthcare-4530592-supplementary.pdf]

Supplementary Table S1. Pulmonary function test grades and definitions.

Grade	Definition
A	Three or more acceptable maneuvers are available, with excellent reproducibility of FVC, FEV1, and PEF. The values are suitable for interpretation of pulmonary function.
B	Three or more acceptable maneuvers are available, with adequate reproducibility of FVC, FEV1, and PEF. The values are suitable for interpretation of pulmonary function.
C	Two or more acceptable maneuvers are available, with moderate reproducibility of FVC, FEV1, and PEF. Caution is warranted in the interpretation of pulmonary function.
D	At least one acceptable maneuver is available, but the reproducibility of FVC, FEV1, and PEF is only moderate or cannot be calculated. The values are not suitable for interpretation of pulmonary function.
F	No acceptable maneuvers are available, and reproducibility cannot be calculated. The values are not suitable for interpretation of pulmonary function.

Abbreviations: FVC, forced vital capacity; FEV1, forced expiratory volume in 1 second; PEF, peak expiratory flow.

Supplementary Table S2. Patient Satisfaction Questionnaire.

Category	Question	Very Satisfied (5)	Satisfied (4)	Neutral (3)	Dissatisfied (2)	Very Dissatisfied (1)
**Decentralized Clinical Trial Components**	Q1. Was the out-of-hospital spirometry testing convenient?<br>Q2. Was it easy to communicate with the research team during the study period?<br>Q3. Overall, were you satisfied with the remote monitoring via phone calls and the mobile application (App)?<br>Q4. Are you willing to participate in future clinical trials that include telemedicine components?<br>Q5. Would you be willing to recommend clinical trials with telemedicine components to others?					
**Portable Spirometer**	Q6. Was the training provided for the portable spirometer adequate?<br>Q7. Was the portable spirometer easy to use?<br>Q8. Was the mobile application (App) easy to use?<br>Q9. Was the biweekly spirometry testing frequency using the portable device appropriate?					

Supplementary Table S3. Portable Spirometry Quality.

**Visit**	**Performed *n***	**Grade A**	**Grade B**	**Grade C**	**Grade D**	**Grade F**
D15	23	10 (43.5%)	0 (0.0%)	4 (17.4%)	8 (34.8%)	1 (4.3%)
D29	19	3 (15.8%)	0 (0.0%)	6 (31.6%)	6 (31.6%)	4 (21.1%)
D43	18	5 (27.8%)	1 (5.6%)	4 (22.2%)	5 (27.8%)	3 (16.7%)
D57	16	5 (31.2%)	1 (6.2%)	7 (43.8%)	3 (18.8%)	0 (0.0%)
D71	11	2 (18.2%)	1 (9.1%)	2 (18.2%)	4 (36.4%)	2 (18.2%)
D85	13	4 (30.8%)	1 (7.7%)	2 (15.4%)	5 (38.5%)	1 (7.7%)

Note: Data are presented as *n* (%). "Performed n" denotes the number of participants who completed spirometry at each scheduled visit and served as the denominator for the percentage of each quality grade within the corresponding row.

Supplementary Table S4. Questionnaire scores according to the quality grade of the first performed home spirometry measurement.

**Group**	**n**	**Mean ± SD**	***p* value**
A/B	10	28 ± 8	0.24
C/D/F	11	31.9 ± 6.7	

Note: Participants were classified into the A/B group or the C/D/F group according to the quality grade of their first performed home spirometry measurement. Of the 23 participants who performed spirometry at Day 15, 21 had both an evaluable first-measurement quality grade and available questionnaire data and were included in this analysis (A/B group, $n = 10$; C/D/F group, $n = 11$). Data are presented as mean ± standard deviation. The p value was calculated using Student's t-test.
